# Adsorption of 1,4-phenylene diisothiocyanate onto the graphene oxide sheets functionalized with polydiphenylamine in doped state

**DOI:** 10.1038/s41598-019-48314-x

**Published:** 2019-08-19

**Authors:** M. Baibarac, M. Daescu, S. N. Fejer

**Affiliations:** 1 National Institute of Materials Physics, Laboratory of Optical Processes in Nanostructured Materials, Atomistilor street 405A, Magurele, P.O. Box MG-7, RO77125 Romania; 2 Pro-Vitam Ltd., Muncitorilor street 16, Sfantu Gheorghe, Romania

**Keywords:** Surfaces, interfaces and thin films, Composites

## Abstract

Adsorption processes of 1,4-phenylene diisothiocyanate (PDITC) on two new platforms of the type graphene oxide (GO) sheets and GO layers functionalization with polydiphenylamine (PDPA) are studied by Raman scattering and photoluminescence (PL). An interaction in solid state phase of the two constituents, i.e. PDITC and GO sheets, and a deposition of PDITC onto the PDPA functionalized GO layers, respectively, by the drop casting method, were performed. In the first case, it is shown that interaction in solid state phase of GO with PDITC leads to an intercalation of the organic compound between GO sheets simultaneously with the appearance of the o-thiocarbamate groups, that induces: (i) an enhancement of the PDITC Raman lines situated in the 400–800 and 1000–1300 cm^−1^ spectral ranges, (ii) a change in the ratio between the relative intensities of the two Raman lines peaked at 1585 and 1602 cm^−1^ accompanied by an up-shift in the case of the second line and (iii) a down-shift of the PDTIC PL band from 502 to 491 nm. Using cyclic voltammetry, an electrochemical functionalization of the GO layers with PDPA doped with H_3_PMo_12_O_40_ heteropolyanions takes place, as demonstrated by Raman scattering and FTIR spectroscopy. The presence of the amine groups in the molecular structure of the doped PDPA functionalized GO layers induces a chemical adsorption of PDITC on this platform, when the thiourea groups appear simultaneously with o-thiocarbamate groups. A chemical mechanism is proposed to take place at the interface of the GO sheets and the doped PDPA functionalized GO layers, respectively, with PDITC.

## Introduction

Organic compound 1,4-phenylene diisothiocyanate (PDITC) is one among from the often used coupling agents (cross-linkers) for biological applications performed in the presence of various surfaces modified with amines of the type ethylenediamine^1^, cysteamine^2,3^, 3-amino-propyl(triethoxysilane)^4^ and so on. Applications of PDITC in the field of electrochemical immunosensors, for the detection of markers such as the epidermal growth factor receptor^1^ and Murine double minute 2^2^ in the brain tissue or human plasma, have involved a chemical adsorption of PDITC onto the gold nanoparticles/electrodes surface modified with cysteamine. The high cost of the Au nanoparticles/electrodes is a major drawback for the manufacturing of such platforms at large-scale. In order to overcome this inconvenient, in the present work, the GO sheets and the GO layers functionalized with polydiphenylamine (PDPA) are characterized. In this work, the attention will be focalised on the understanding of the PDITC adsorption mechanism during the interaction in the solid-state phase of PDITC with the GO sheets and the deposition by drop casting method of PDITC onto the GO layers functionalized with PDPA. A study concerning the adsorption/interaction of the PDITC on/with GO sheets or their composites has not been reported until now. In this work, the main aim consists in the understanding of the adsorption mechanism of PDITC onto the GO sheets functionalized with PDPA in doped state. Thiocyanate adsorption was studied using Ag nanoparticles^5^, Au films modified with cysteamine^6^ and the Au-Pd core-shell nanoparticles^7^. Optical methods often used in evaluating PDITC adsorption on the metallic nanostructures include surface enhanced Raman scattering, IR spectroscopy and atomic force microscopy^5,6^.

In this work, the Raman scattering will also be used for assessing the PDITC adsorption on the GO sheets and the PDPA functionalized GO layers. The photosensitivity of PDITC will be highlighted by PL in all subsequent studies shown in this work, in order to take into account this process. The influence of GO on the PDITC photosensitivity will also be shown. A mechanism for the PDITC adsorption on the two structures, i.e. the GO sheets and the PDPA functionalized GO layers, will be reported as well.

## Results and Discussions

### Optical properties of the GO sheets interacting with PDTIC

**Figure 1:**
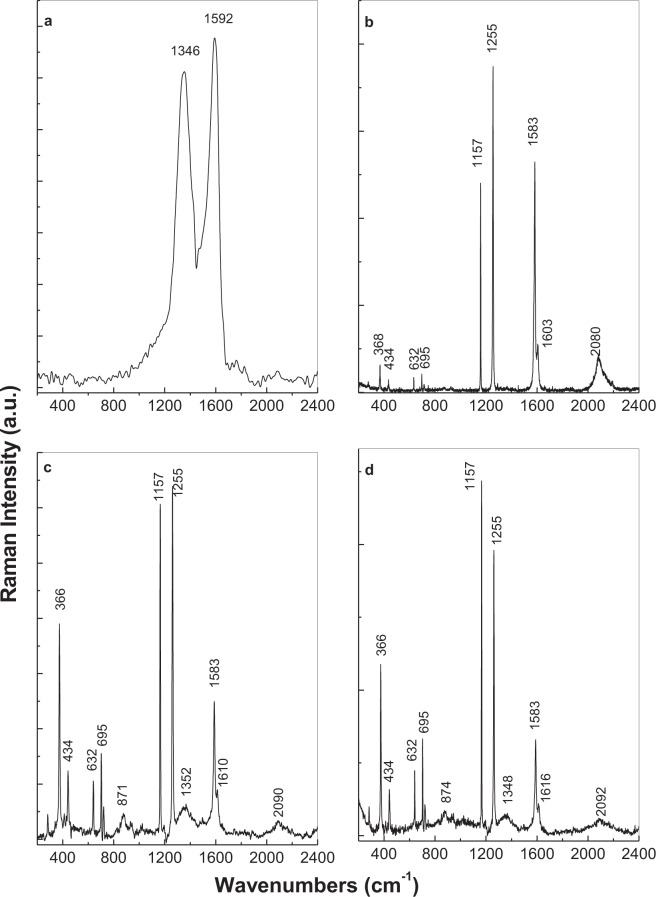
Raman spectra of GO sheets (**a**), PDITC (**b**) and platelets of PDITC with 1 wt.% GO (**c**) and 2 wt.% GO (**d**).

Figure 1a shows the Raman spectrum of GO, which is characterized by two bands peaked at 1346 and 1592 cm^−1^, assigned to the hexagonal rings breathing vibrational mode and the E_2g_ phonon mode at the Brillouin zone centre^8^. Figure 1b shows the Raman spectrum of PDITC, characterized by the three high intensity lines situated in the 1000–1650 cm^−1^ spectral range and other five lines of low intensity localized in the 300–700 and 2000–2200 cm^−1^ spectral ranges. The PDITC Raman lines, peaked at 368, 434, 632–695, 1157, 1257, 1583, 1603 and 2080 cm^−1^, are assigned to the following vibrational modes: deformation of a p-substituted benzene ring^9^, bending deformation of the NCS bond^10^, asymmetric C-S stretching^11^, C-S bending^11^, C-H in benzene ring + C-C stretching + C-N stretching^9^, C=C + C-C stretching in benzene ring^9,12^, C-C stretching + C-H bending in benzene ring^9^ and C=N stretching^5^, respectively. The following differences are observed in the Raman spectra of the platelets of PDITC with 0, 1 and 2 wt.% GO (Fig. 1b–d): (i) an enhancement in the relative intensities of the PDITC Raman lines situated in the 400–800 and 1000–1300 cm^−1^ spectral ranges, when the concentration of GO in the PDITC platelets weight is equal to 1 and 2 wt.% (Fig. 1c,d); (ii) a decrease in the ratio between the relative intensities of the two Raman lines peaked at 1583 and 1603–1616 cm^−1^ (I_1583_/I_1603–1616_) from 5.29 (Fig. 1b) to 2.82–2.97 (Fig. 1c,d) with increasing the GO concentration in the PDITC platelets weight; this change is accompanied of an up-shift of the Raman line assigned to the vibrational mode of C-C stretching +C-H bending in benzene ring, from 1603 to 1616 cm^−1^, when the GO concentration in the PDITC platelets weight increases from 0 to 2 wt.%; iii) the ratio between the relative intensities of the Raman lines peaked at 1583, 1255–1257 and 1157 cm^−1^ (I_1583_/I_1255–1257_ and I_1583_/I_1157_) decrease from 0.71 and 1.1 (Fig. 1b) to 0.39 and 0.41 (Fig. 1c) or 0.34 and 0.27 (Fig. 1d), as the GO concentration increases from 0 to 1 and 2 wt.%; iv) a significant decrease in the ratio between the relative intensities of the Raman lines peaked at 1583 and 366–368 cm^−1^ (I_1583_/I_366–368_) from 8.82 (Fig. 1b) to 0.63 (Fig. 1c) and 0.56 (Fig. 1d), when the GO concentration in the PDITC platelets weight increases from 0 to 2 wt.%; and v) a gradual up-shift of the D band of GO from 1346 cm^−1^ (Fig. 1a) to 1348 cm^−1^ (Fig. 1c) and 1352 cm^−1^ (Fig. 1d) occurs in the presence of PDITC.

All these changes indicate the presence of a steric hindrance effect that can be explained only if we accept that an interaction between PDITC and GO takes place according to Fig. 1S. Figure 1S indicates the formation of two compounds, the former corresponding to the GO sheets intercalated with PDITC (GO-PDITC-GO) which shows o-thiocarbamate groups and a second one which illustrates the GO sheets modified with PDITC (GO-PDITC) having a molecular structure which contains both of the o-thiocarbamate functional groups and the isothiocyanate terminated surface. The formation of the GO-PDITC-GO compound explains the enhancement of the PDITC Raman lines situated in the 400–800 and 1000–1300 cm^−1^ spectral ranges.

**Figure 2:**
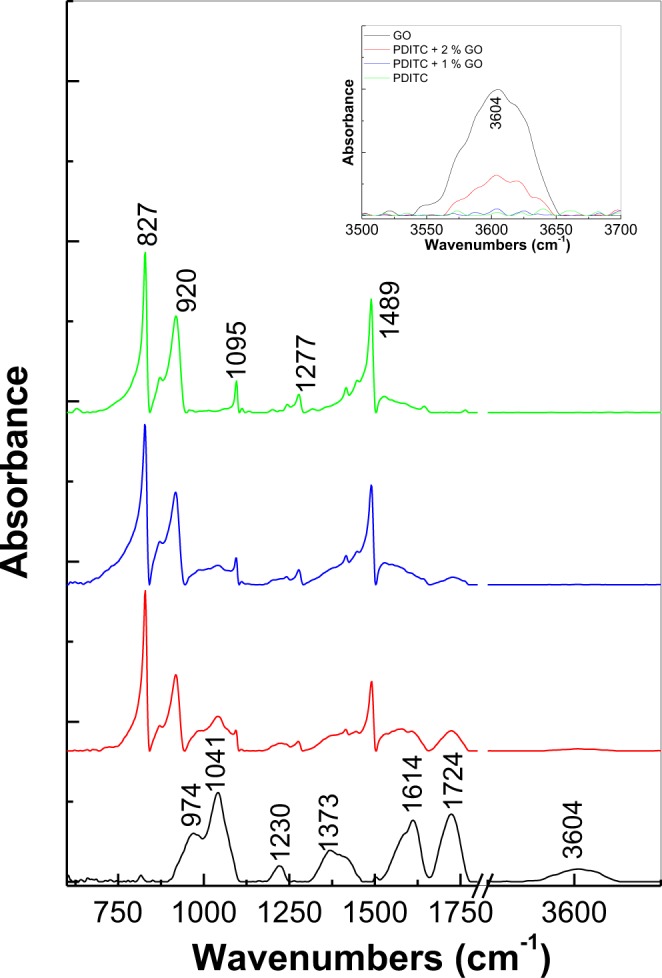
IR spectra of GO sheets (black curve), PDITC (green curve) as well as platelets of PDITC with 1 wt.% GO (blue curve) and 2 wt.% GO (red curve).

**Figure 3:**
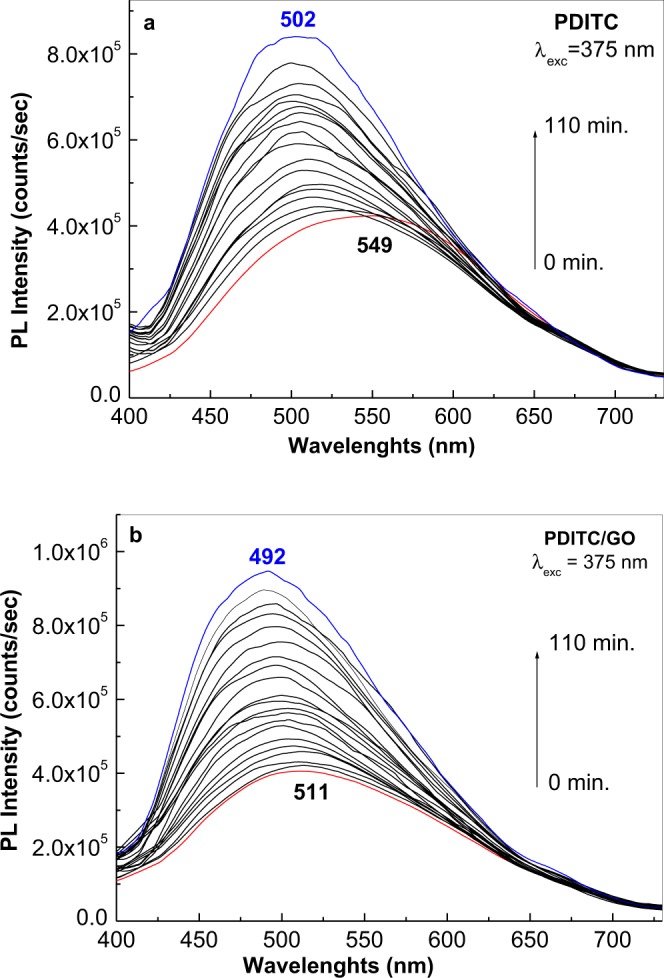
PL spectra of PDITC (**a**) and GO sheets interacting with PDITC (**b**). Red and blue curves correspond to the PL spectra in the initial state and after irradiation under the excitation wavelength of 375 nm for 110 minutes. Black curves correspond to the PL spectra measured after a gradual increase of the irradiation time from 0 to 110 min.

Additional information concerning the interaction of the GO sheets with PDITC is obtained by IR spectroscopy. Figure 2 shows the IR spectra of PDITC and the GO sheets before and after their interaction in solid state phase. According to Fig. 2, the main IR bands of the GO sheets are peaked at 1041, 1230, 1373, 1614, 1724 and 3604 cm^−1^, these being assigned to the following vibrational modes: C=C, OH groups from the GO sheet edges, alkoxy groups, H_2_O adsorption onto the GO sheets' surface, C=O groups belonging COOH and OH stretching^8^. The most intense IR bands of PDITC are peaked at 827 and 1489 cm^−1^, these being assigned to the vibrational modes of bending of C-H out of plane in benzene p-substituted and –C=N-benzene ring, respectively^9,13^. The interaction of PDITC with the GO sheets leads to the following changes in the IR spectra of the two constituents: (i) a decrease in the ratio between the absorbance of the two IR bands of PDITC peaked at 1489 and 827 cm^−1^ from 0.71 (green curve in Fig. 2) to 0.43 (red curve in Fig. 2) and (ii) an increase of the ratio between the absorbance of the IR bands belonging to the GO sheets peaked at 1724 and 3604 cm^−1^, from 5.4 (green curve in Fig. 2) to 9.84 (blue curve in Fig. 2) and 23 (red curve in Fig. 2) simultaneously with a more pronounced decrease in the absorbance of the IR band at 3604 cm^−1^. The decrease of the absorbance of the IR band at 1489 cm^−1^ indicates a smaller weight of the –C=N-benzene ring vibrational mode after the interaction of the GO sheets with PDITC, this being in good agreement with the processes shown in Fig. 1S. In addition to above changes, a down-shift of the PDTIC PL band from 549 nm to 511 nm is observed, as seen in Fig. 3. An interesting fact shown in Fig. 3 is the change of the PL spectra of PDITC and that of the GO sheets intercalated with PDITC, when the excitation wavelength is equal with 375 nm. When increasing the irradiation time to 110 min., a gradual increase is observed in the relative intensity of the PL bands of PDITC and the GO sheets intercalated with PDITC, from 423.532 and 405.404 counts/sec to 840.388 and 947.136 counts/sec, respectively. This change is accompanied by a down-shift of the PL band maximum of PDITC and the GO sheets intercalated with PDITC from 549 and 511 nm at 502 and 492 nm, respectively. In our opinion, these variations indicate a photochemical process, induced by water vapours form air, as shown in Fig. 2S for PDITC. A similar reaction can be invoked for the GO-PDTIC compound, formed through the interaction of PDITC with GO according to Fig. 1S. PL spectra remained unchanged for four hours, when recorded in vacuum (at the pressure of 5.4 × 10^−6^ mbar). The difference in the value of the PL band maximum shift is due to the small number of the isothiocyanate groups in the compounds resulted from the interaction of the GO sheets with PDITC. Summarizing all these results, we can conclude that: i) interaction in solid state phase of GO with PDITC leads to an intercalation of the organic compound between GO sheets, leading to the generation of new o-thiocarbamate functional groups, and ii) the manipulation of PDITC and its derivates must to be carried out in the absence of UV light in order to avoid occurrence of photochemical reactions.

### Optical properties of GO sheets functionalized with PDPA before and after the PDITC deposition

**Figure 4:**
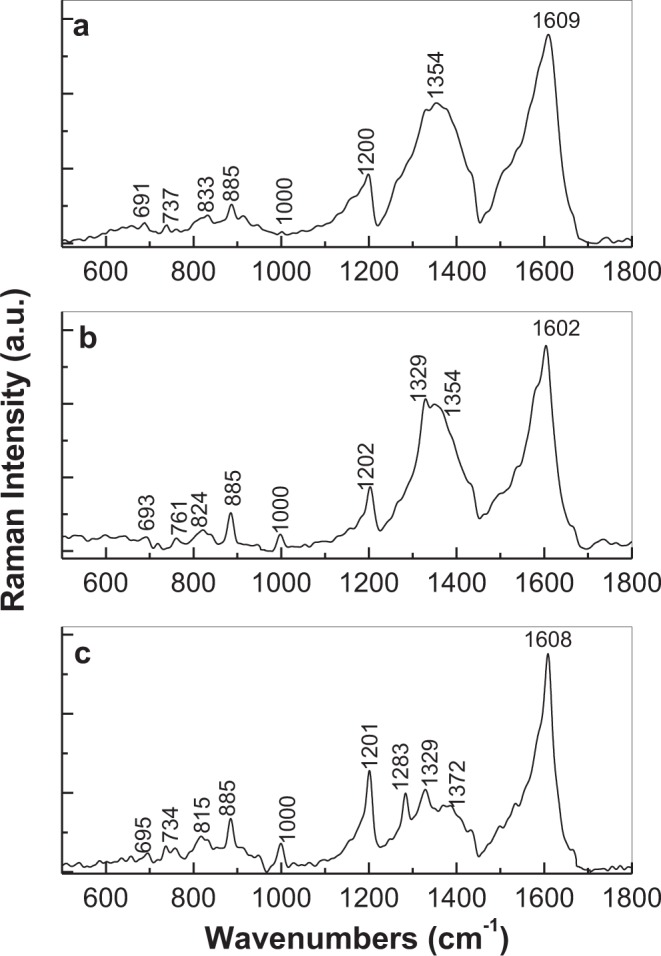
Raman spectra of the GO layers electrochemical functionalized with PDPA, when SPCE was immersed into the semi-aqueous solution of 10^−3^ M H_3_PMo_12_O_40_ and 1 M HCl in DMF: H_2_O with different concentration of DPA, i.e.10^−3^ (**a**), 5 10^−3^ (**b**) and 10^−2^ M (**c**).

**Figure 5:**
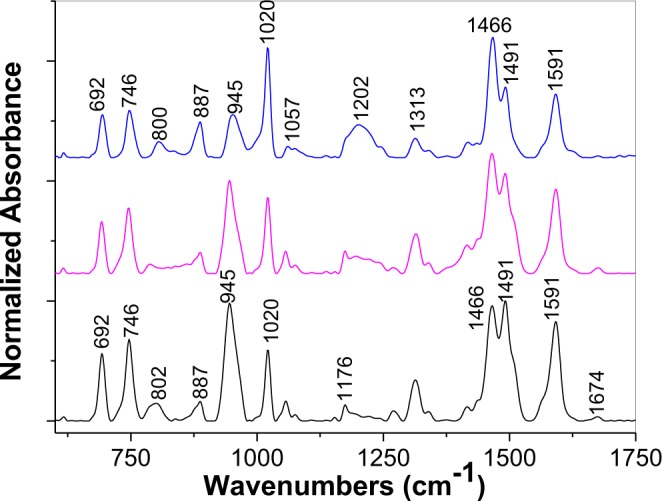
IR absorption spectra of the GO layers electrochemically functionalized with PDPA, when SPCE was immersed into the semi-aqueous solution of 10^−3^ M H_3_PMo_12_O_40_ and 1 M HCl in DMF: H_2_O with different concentrations of DPA, i.e.10^−3^ (blue curve), 5 × 10^−3^ (magenta curve) and 10^−2^ M (black curve).

Figure 4 shows the Raman spectra of the GO sheets electrochemically functionalized with PDPA, when SPCE was immersed into the semi-aqueous solutions of 10^−3^ M H_3_PMo_12_O_40_ and 1 M HCl in DMF:H_2_O with different concentrations of DPA, i.e.10^−3^ (Fig. 4a), 5 10^−3^ (Fig. 4b) and 10^−2^ M (Fig. 4c). A short comment concerning the main Raman lines of H_3_PMo_12_O_40_ and PDPA doped with H_3_PMo_12_O_40_ heteropolyanions is necessary at this stage. According to the study reported by Tatibouet *et al*., the Raman spectrum of H_3_PMo_12_O_40_ is characterized by an intense line with maximum at 1000 cm^−1^ having a shoulder at 996 cm^−1^, which were assigned to the symmetrical and anti-symmetrical vibrational mode of the Mo=O bond^14^. Five lines were reported in the Raman spectra of the PDPA in doped state, these being peaked at 1176, 1201, 1368, 1541 and 1603 cm^−1^ ^15,16^. These Raman lines were attributed to vibrational modes of C-H bending in benzene ring, C-N stretching, semi-quinoid radical structure, C=C stretching in quinoid ring and C-C stretching in benzene ring, respectively^9,15–17^. The low intensity Raman lines at 691 and 737 cm^−1^ were assigned to the inter-ring and benzene ring deformation vibrational modes of PDPA^9^. In comparison with the Raman spectra of GO and PDPA, by increasing the DPA concentration in the synthesis solution of the GO sheets electrochemically functionalized with PDPA, in Fig. 4 one observes: (i) an increase in the intensity of the Raman lines peaked at 885 and 1000 cm^−1^; the Raman line with the maximum at 885 cm^−1^ belongs to the MoO_3_ vibrational mode^14^; (ii) a down-shift of the Raman line from 833 to 815 cm^−1^ accompanied of a change of the ratio between the relative intensity of Raman lines localized at 1602–1609 and 1200–1202 cm^−1^ from 3.2 to 2.3; and (iii) a gradual decrease in the relative intensity of the D band of GO simultaneously with the appearance of the Raman lines peaked at 1283, 1329 and 1372 cm^−1^. These changes indicate: (i) the insertion of the H_3_PMo_12_O_40_ heteropolyanions onto the PDPA macromolecular chain, confirmed by the presence of the Raman lines situated in the 850–1100 cm^−1^ spectral range; (ii) a progressive coverage of the GO sheets with PDPA in the doped state by the intensity increase of Raman lines of the macromolecular compound and (iii) the covalent functionalization of the GO sheets with PDPA, highlighted by the new Raman line peaked at 1283 cm^−1^, that was also reported in the case of triphenylamine and its derivate compounds, this being assigned to the vibrational mode of large C-N stretches with associated C-C stretches^18^. The molecular structures of the GO sheets covalently functionalized with PDPA doped with the H_3_PMo_12_O_40_ heteropolyanions as well as of the macromolecular compound in doped state are shown in Fig. 3S. As observed in Fig. 3S, the following functional groups of GO are shown schematically: hydroxyl, carboxyl and ether. In order to better understand these molecular structures, Fig. 4S shows the reaction of PDPA doped with the H_3_PMo_12_O_40_ heteropolyanions with the GO sheets. Additional information concerning the functionalization process is shown in Fig. 5 by IR spectroscopy. In all three cases, the IR spectra are characterized by bands belonging to PDPA and the H_3_PMo_12_O_40_ heteropolyanions. According to previous studies^19,20^, the IR bands belonging to H_3_PMo_12_O_40_ have the maxima peaked at 792, 870, 960 and 1065 cm^−1^. A careful analysis of Fig. 5 highlights that the first two IR bands, assigned to the Mo-O-Mo vibrational modes, are up-shifted from 792 and 870 cm^−1^ to 800 and 887 cm^−1^, while the last two IR bands attributed to the Mo=O and P-O vibrational modes are down-shifted from 960 and 1065 cm^−1^ to 945 and 1057 cm^−1^ ^19,20^. The change of the position of these IR bands was correlated with the doping level induced by this heteropolyacid to conjugated polymers^20^. In this context, we note that this behaviour is similar to that reported in the case of the single-walled carbon nanotubes functionalized with PDPA doped with H_3_PMo_12_O_40_ heteropolyanions^16^. The IR bands belonging to PDPA are peaked at 692, 746, 1020, 1176–1202, 1313, 1466, 1491 and 1591 cm^−1^ and their assignment is shown in Table 1S^9,21^. As increasing the DPA concentration in the synthesis solution, an increasing in the absorbance of the IR band peaked at 1591 cm^−1^ is noted as a consequence of a greater weight of PDPA compared to the GO layer surface. As shown in our previous work, the ratio between the absorbance of the IR bands of standalone PDPA peaked at 698, 752 and 1028 cm^−1^ (A_698_/A_1028_ and A_698_/_1028_) is equal to 7.1 and 6.9, respectively^21^. These IR bands were assigned to the vibrational modes of inter-ring deformation, benzene ring deformation and A_1_ benzene^9,21^. According to Fig. 5, in the case of the SPCE modified with a GO layer as the DPA concentration in the synthesis solution increases from 10^−3^ M to 5 10^−3^/10^−2^ M, one observes that: (i) the vibrational modes of inter-ring deformation, benzene ring deformation and A_1_ benzene are down-shifted at 692, 746 and 1020 cm^−1^; (ii) the values of the A_692_/A_1020_ and A_746_/_1020_ ratios are changed from 0.39 and 0.43 to 0.68/0.95 and 0.86/1.15, respectively and (iii) the ratio between the absorbances of the IR bands peaked at 945 and 1020 cm^−1^ is changed from 0.39 to 1.23 and 1.67. The higher absorbance of the IR bands assigned to the deformation vibrational modes of the benzene and quinoid rings as well as inter-rings highlights the significant steric hindrance effects induced by the covalent bonding of PDPA doped with H_3_PMo_12_O_40_ heteropolyanions onto the GO layers.

**Figure 6:**
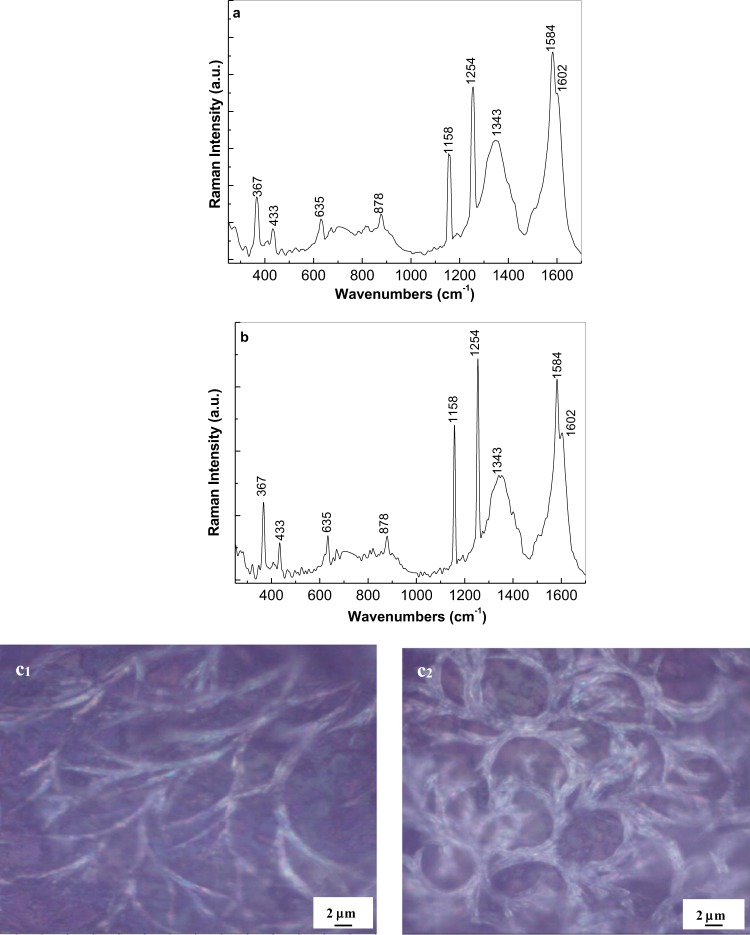
Raman spectra of the GO layers electrochemically functionalized with PDPA after the deposition of PDITC from a solution of PDITC in C_2_H_5_OH having the concentration 0.1 mg/ml, by the evaporation of 1 ml (**a**) and 2 ml (**b**) solvent. Figures c1 and c2 show the optical image of the GO layers electrochemically functionalized with PDPA after the deposition of PDITC by the evaporation of 1 ml and 2 ml C_2_H_5_OH, respectively.

**Figure 7:**
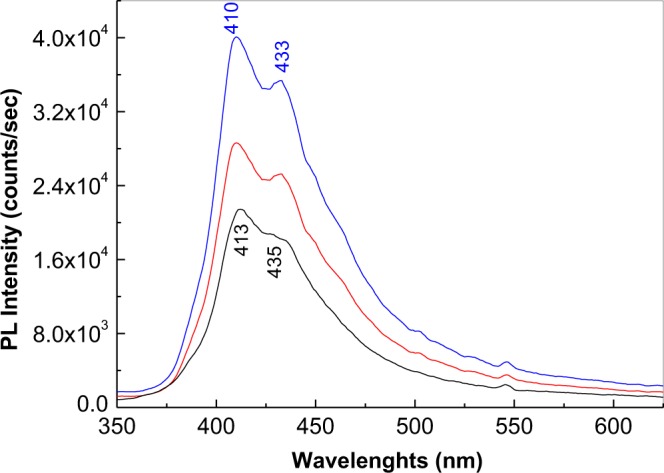
PL spectra of the GO layers electrochemically functionalized with PDPA before (black curve) and after the deposition of PDITC from a solution of PDITC in C_2_H_5_OH having the concentration of 0.1 mg/ml, by the evaporation of 1 ml (red curve) and 2 ml solvent (blue curve) recorded under the excitation wavelength of 275 nm.

Figure 6 shows the Raman spectra of the GO layers covalently functionalized with PDPA in doped state, after the deposition of PDITC from a solution of PDITC in C_2_H_5_OH having the concentration 0.1 mg/ml, by the evaporation of 1 ml (a) and 2 ml (b) solvent. The GO layers electrochemical functionalized with PDPA in doped state were prepared using a semi-aqueous solution of 10^−3^ M DPA, 10^−3^ M H_3_PMo_12_O_40_ and 1 M HCl in DMF: H_2_O. In the two cases, the Raman spectra are dominated by the PDITC Raman lines. Other Raman lines observed in Fig. 6 are those peaked at 878 and 1343 cm^−1^ belonging to the Mo-O-Mo vibrational modes of the H_3_PMo_12_O_40_ heteropolyanions and the GO D band. The absence of the Raman lines of the PDPA is difficult to be understood. In order to show an evidence for the presence of macromolecular compounds, Fig. 7 depicts the PL spectra of the GO layers covalently functionalized with PDPA in doped state before and after the PDITC deposition. The PL spectrum of the GO layers covalently functionalized with PDPA in doped state, recorded under the excitation wavelength of 275 nm, is characterized by a complex emission band having the maximum at 413 nm. After the PDITC deposition, only an increase in the relative intensity of the PL spectrum of the GO layers covalently functionalized with PDPA in doped state is observed. This increase in the relative intensity of the PL spectrum of the GO layers covalently functionalized with doped PDPA in the presence of PDITC may have originated in the emergence of new luminescent centres. In this context, we note that the presence of the amine groups in the molecular structure of the doped PDPA functionalized GO layers, can induce a chemical adsorption of PDITC on this platform, when the thiourea groups appear simultaneously with those of the type o-thiocarbamate. Figure 5S shows the reaction of PDITC with the doped PDPA functionalized GO layers. A similar activation of amino groups of the working electrode by interaction with PDITC was reported in the case of: (i) cysteamine modified Au electrode^2^, (ii) 11-Mercaptoundecanoic acid modified Au electrode^22^; and (iii) β-cyclodextrin – reduced graphene oxide-tetraetylene^23^. An optical image of the samples of the doped PDPA functionalized GO layers after the PDITC adsorption is shown in Figure 6c_1_ and 6c_2_.These images highlight the formation of a one-dimensional macrostructures as a consequence of the generation of the thiourea groups by the PDITC adsorption onto the doped PDPA functionalized GO layers. The presence of isothiocyanate groups onto the surface of the GO sheets and that of the GO layers functionalized by PDPA, which were intercalated/modified with PDITC, open new perspectives on the applications of these platforms in the immunosensors field for the detection of the cancer markers, for example.

**Figure 8:**
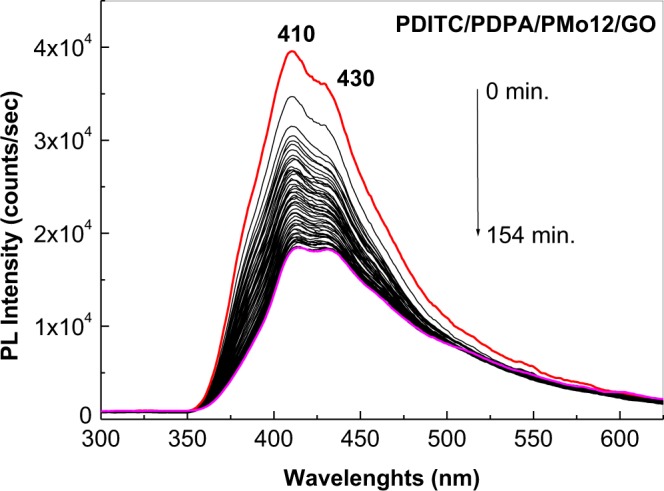
Evolution of PL spectra of the PDITC/PDPA/GO sample, recorded under the excitation wavelength of 275 nm, time of 154 min. Red curve corresponds to the emission spectrum before of UV irradiation of the PDITC/PDPA/GO sample. Black curves correspond to each PL spectra successively collected after 185 seconds of the UV irradiation. Magenta curve corresponds to the PL spectrum of the PDITC/PDPA/GO sample after 154 min. of UV irradiation.

We note that samples resulted after the deposition of PDITC onto the GO layers functionalized by PDPA must be handled in the absence of UV light. In order to support this sentence, Fig. 8 shows the evolution of PL spectra of the sample obtained after the deposition of PDITC from a solution of PDITC in C_2_H_5_OH having the concentration of 0.1 mg/ml, by the evaporation of 2 ml solvent onto the GO sheets functionalized with PDPA doped with H_3_PMo_12_O_40_ heteropolyanions. According to Fig. 8, under the excitation wavelength of 275 nm, one observes a gradual decrease in the intensity of the PL band of the PDITC/PDPA/GO sample from 39.549 counts/sec to 18.477 counts/sec after 154 min. of UV irradiation. This behaviour is different of that reported in Fig. 3. In our opinion, the photochemical process which occurs in the case of the PDITC/PDPA/GO sample in the presence of UV light can be described by the chemical reaction shown in Fig. 6S.

## Conclusions

In this work, we have reported new results concerning the adsorption of PDITC onto two platforms, i.e. GO sheets and GO layers covalently functionalized with PDPA in doped state. Our results have highlighted that:

i) the interaction in solid state phase of PDITC with the GO sheets results in a chemical adsorption of organic compound onto the carbon nanostructure. Taking into account the changes reported in the Raman spectra of PDITC and the GO sheets, two resulting compounds were identified: one corresponding to the GO sheets intercalated with PDITC, exhibiting o-thiocarbamate groups, and a second in which the GO sheets are modified with PDITC having in the molecular structure both the o-thiocarbamate functional groups and an isothiocyanate terminated surface.
ii) the electropolymerization of DPA in the presence of the semi-aqueous solution of 10^−3^M H_3_PMo_12_O_40_ and 1 M HCl in DMF: H_2_O, when SPCE modified with GO was used as working electrode, leads to the GO layers covalently functionalized with PDPA in doped state;
iii) the adsorption of PDITC onto the GO layers covalently functionalized with PDPA in doped state involves the appearance of the thiourea groups simultaneously with those of the type o-thiocarbamate.
iv) a photochemical process was reported in the case of the PDITC adsorption onto the GO sheets surface as well as the GO layers covalently functionalized with PDPA in doped state. These results indicate the necessity of the manipulation of these platforms in the absence of the UV light.

## Methods

All chemical compounds, i.e. diphenylamine (DPA), H_3_PMo_12_O_40_ x H_2_O_,_ HCl, graphite, dimethylformamide (DMF), C_2_H_5_OH, sodium nitrate, H_2_SO_4_, KMnO_4_ and H_2_O_2_ were used as received from Sigma-Aldrich. PDITC was purchased from Expert Trade. The GO sheets were synthetized according to ref.^8^.

In the present work, H_3_PMo_12_O_40_ acts as an initiator for the growth of macromolecular chain of PDPA onto the GO sheets and a doping agent of PDPA. The GO layers functionalized with PDPA doped with H_3_PMo_12_O_40_ heteropolyanions were prepared by electrochemical polymerization of DPA onto the screen-printed carbon electrodes (SPCE) modified with GO purchased from DropSens. The cyclic voltammetry studies were carried out by the immersion of the SPCE into a solution consisting from DPA (10^−3^, 5 10^−3^ or 10^−2^ M), 10^−3^ M H_3_PMo_12_O_40_ and 1 M HCl in semi-aqueous solution of DMF:H_2_O having the volumetric ratio of 1:1. The potential range was between +100 and +960 mV vs. Ag electrode and a sweep rate equal with 50 mV s^−1^ was used. The reported cyclic voltammograms using SPCE were recorded with a potentiostat/galvanostat, Voltalab 80 model, purchased from Radiometer Analytical.

The interaction in solid state phase of PDITC with the GO sheets was carried out in the absence of light by the mechanico-chemical reaction of the two constituents that were compressed non-hydrostatically at the pressure of 0.58 GPa, time of 5 minutes, resulting in platelets of PDITC with 1 and 2 wt.% GO.

The PDITC deposition on the PDPA functionalized GO layers was performed by the drop casting method using a solution of PDPA in C_2_H_5_OH having the concentration of 1 mg/ml.

Raman spectra of PDTIC, GO sheets, PDPA intercalated GO sheets and PDPA functionalized GO layers before and after the interaction with PDTIC were recorded under the excitation wavelength of 514 nm using a spectrophotometer Raman, T64000 model, from Horiba Jobin Yvon, endowed with an Ar laser.

The PL spectra of PDTIC, PDPA intercalated GO sheets and PDPA functionalized GO layers before and after the interaction with PDTIC were recorded at room temperature, under the excitation wavelength of 375 nm and 275 nm, respectively, with a spectrophotometer Fluorolog-3.2.2.1, from Horiba Jobin Yvon.

The IR spectra of GO sheets before and after the interaction with PDITC were recorded with a FTIR spectrophotometer, Vertex 80 model, from Bruker.

## Supplementary information

Supplementary Information
