# Supplementary material for: Adsorption of 1,4-phenylene diisothiocyanate onto the graphene oxide sheets functionalized with polydiphenylamine in doped state
Source: Sci Rep. 2019 Aug 19;9:11968. doi: 10.1038/s41598-019-48314-x (PMC6700158; doi:10.1038/s41598-019-48314-x)
Supplement: Supplementary file 1 — Supplementary Information [file 41598_2019_48314_MOESM1_ESM.docx]

**Supplementary Information**

Adsorption of 1,4-phenylene diisothiocyanate onto the graphene oxide sheets functionalized with polydiphenylamine in doped state

M. Baibarac^1*^, M. Daescu^1^, and S.N. Fejer^2^

^1^Laboratory of Optical Processes in Nanostructured Materials, National Institute of Materials Physics, Bucharest, P.O. Box MG-7, R077125, Romania

^2^Pro-Vitam Ltd., Muncitorilor street 16, Sfantu Gheorghe, Romania

*E-mail : barac@infim.ro

o-thiocarbamate functional group

**Figure 1S** The interaction of the GO sheets with PDITC.

**Figure 2S** The photochemical reaction of PDITC in the presence of the water vapours from air

**a)**


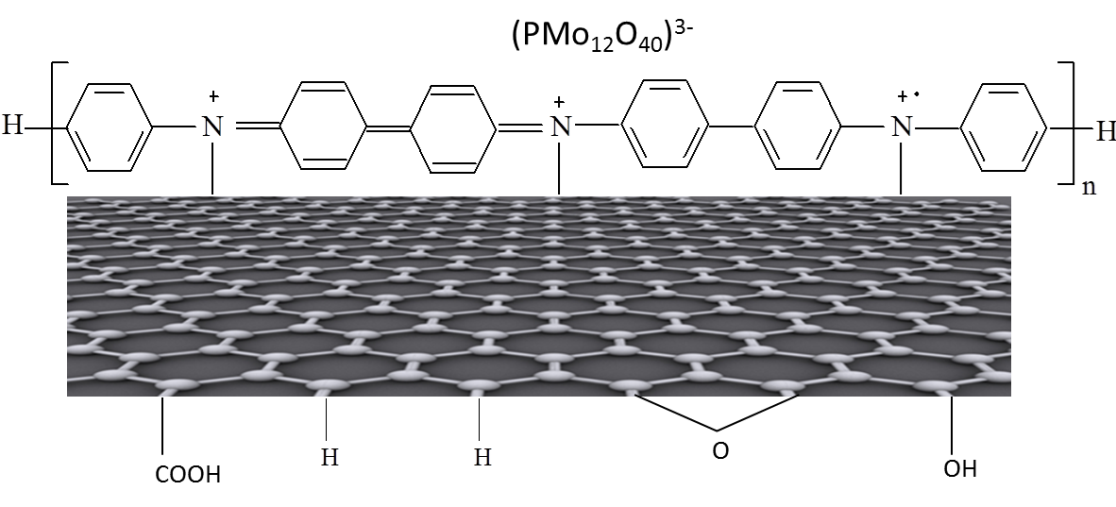


**GO/PDPA^3+^(PMo_12_O_40_)^3-^**


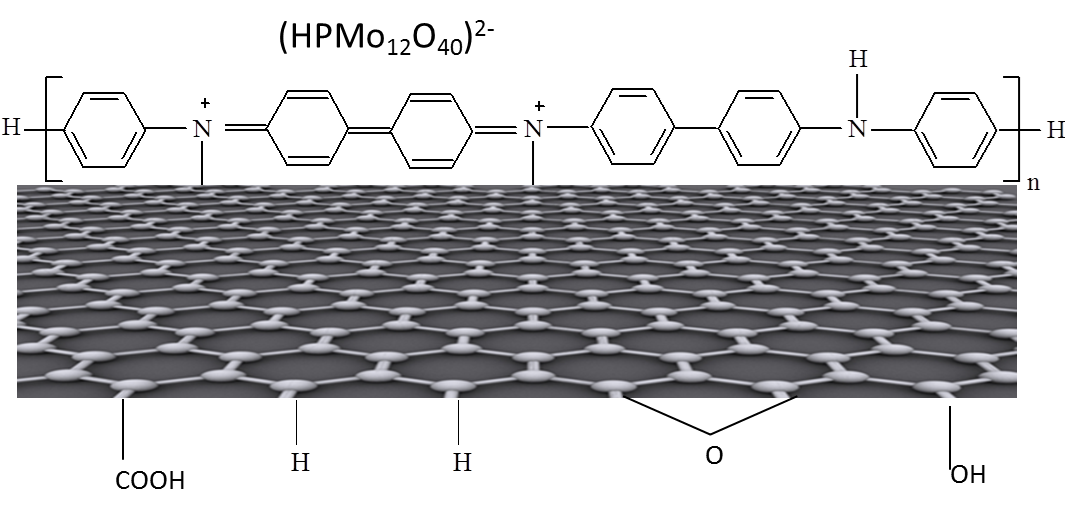


**GO/PDPA^2+^(HPMo_12_O_40_)^2-^**

**b)**

**Figure 3S** The molecular structures of: a) the GO sheets covalently functionalized with PDPA doped with the PMo_12_O_40_^3-^ and HPMo_12_O_40_^2-^ heteropolyanions as well as b) PDPA doped with the heteropolyanions of the type X^3-^ = PMo_12_O_40_^3-^ and X^2-^ = HPMo_12_O_40_^2-^.

**Figure 4S** Reaction of PDPA doped with the H_3_PMo_12_O_40_ heteropolyanions with the GO sheets


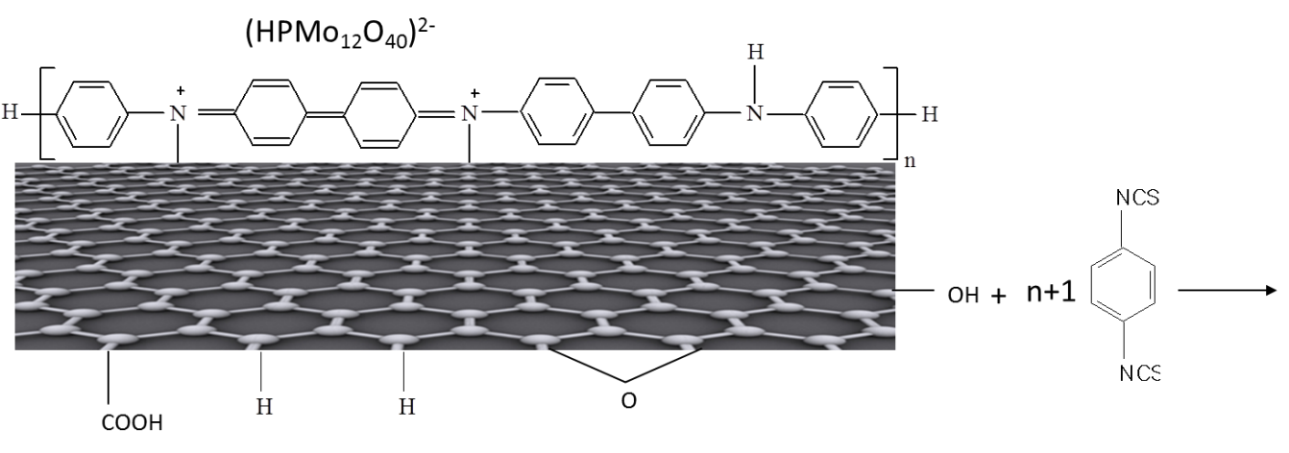


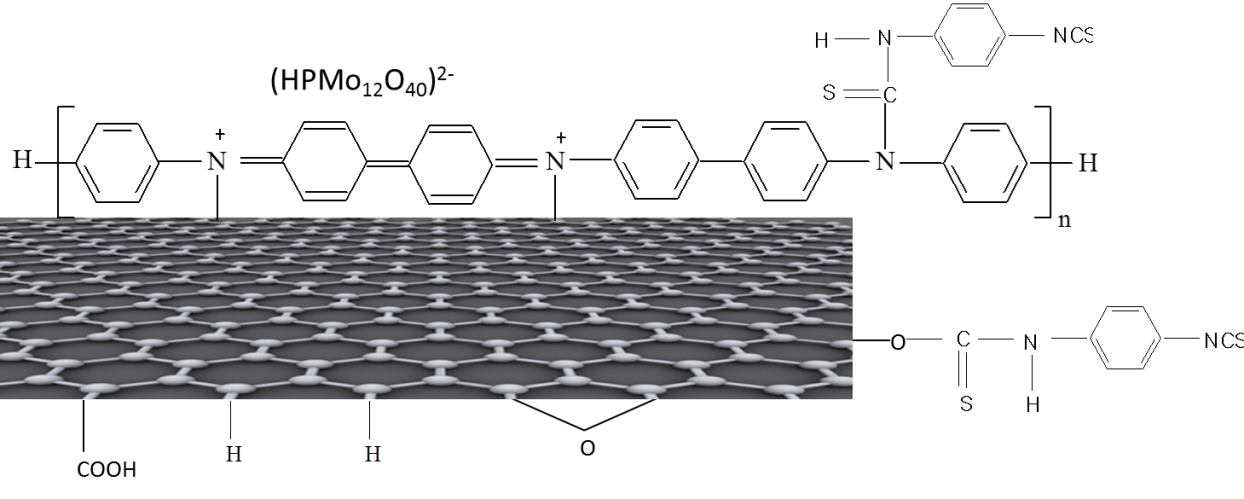

Structure of thiourea derivates:

R-N=C=S

Structure of isothiocyanate derivates:

R_3_ = C_6_H_4_-NCS; R_2_ = C_6_H_5_, R_1_ = GO[PDPA^2+^(HPMo_12_O_40_)^2-^] (OCSNHC_6_H_4_NCS)

Thiourea group: -NH-CS-NH- ; Isothiocyanate group: - N=C-S

**Figure 5S**. The reaction of PDITC with the doped PDPA functionalized GO layers

**Figure 6S**. The reaction of the doped PDPA functionalized GO layers and covered with PDITC in the presence of the UV light

**Table 1S.** The vibrational modes of PDPA active in IR spectroscopy

| **ν (cm^-1^)** | **Assignements^9, 21^** |
| --- | --- |
| 692 | Inter-rind deformation |
| 746 | Benzene ring deformation |
| 1020 | Quinoid ring deformation |
| 1176 | C-H bending in quinoid ring |
| 1202 | C-H bending in benzene ring |
| 1313 | C-C stretching + C-H deformation in benzene ring |
| 1466 | C=N stretching + C-H bending in benzene ring |
| 1491 | C=N stretching |
| 1591 | C-C stretching in benzene ring + C=C stretching in quinoid ring |
| 1674 | C-C stretching in -NH^+^=Quinoid ring=Quinoid ring=NH^+^- |
